# P057 Temporal arteritis without giant cell arteritis

**DOI:** 10.1093/rap/rkag113.087

**Published:** 2026-11-03

**Authors:** Kieran Kitchener, Alfred Rone, Muhammad Sharif, Adrian Peall

**Affiliations:** Hereford County Hospital, Hereford, United Kingdom; Hereford County Hospital, Hereford, United Kingdom; Hereford County Hospital, Hereford, United Kingdom; Hereford County Hospital, Hereford, United Kingdom

## Abstract

**Introduction:**

Literature contains few cases of overlap between GCA and GPA, with variations in the time between presentations of each, in our case a patient presented with symptoms of temporal arteritis alongside renal dysfunction, and was found to have an ANCA vasculitis with evidence of temporal arteritis.

This is an uncommon presentation, and accurate diagnosis can make a significant difference to ensuring a patient receives the correct management for their disease.

**Case description:**

A 60-year-old woman, with a history of unilateral nephrectomy for renal cancer and a diagnosis of chronic fatigue syndrome, presented with a three-month history of lethargy, muscle pains, anorexia headaches, jaw claudication and nausea, with symptoms starting insidiously and progressing over time. She had initially thought all symptoms were related to chronic fatigue and so had not acted upon them until they progressed.

A urine dip was positive for blood and protein with her GP and was referred in to hospital for further review. Blood tests revealed a raised CRP of 266 and a white cell count of 12.3. Over the next few days of admission, she developed a worsening acute kidney injury, with creatinine increasing from a baseline of 88mmol/l to a peak of 279mml/l ten days later.

Examination found a single splinter haemorrhage in her left middle finger, but this had a good probability of being related to mild trauma. There was some mild tenderness to the scalp, but this had eased on the use of intravenous paracetamol. Other examination findings were non-specific, with mild epigastric tenderness and minimal basal crackles to the lungs.

A temporal artery ultrasound was requested whilst further blood tests including ANA and ANCA were pending. The ultrasound showed an echolucent halo in the left parietal branch of the temporal artery. Prednisolone was started with a rapid improvement in her symptoms. PR3 then returned later with raised level of 49.

She was discussed with the local nephrology team for consideration of a biopsy, but this was not performed due to the solitary kidney.

She was continued on the high dose weaning regime of prednisolone and was transferred to the renal centre for rituximab. Between these she had a good response to treatment and results normalised and she is currently weaning down in dose.

**Discussion:**

On first review and if she had only presented to day cases without further review, it would have been easy to diagnose this lady with giant cell arteritis based on the findings of the ultrasound. It was only through the fact that she had a hospital stay with monitoring of renal function and its deterioration that an ANCA-associated vasculitis was diagnosed instead of GCA.

For us, this raises the issues around whether we are risking missing cases where an individual is presenting with symptoms of temporal arteritis and an alternative underlying diagnosis may be present.

On review of literature, there are several cases of patients presenting with symptoms of giant cell arteritis and are later diagnosed with a form of ANCA associated vasculitis, but few where the two are seen simultaneously. It is unclear if this is due to one presenting before the other, or just because there was insufficient investigation at the time to diagnose this.

Treatment with steroids alone would have been unlikely to fully control an ANCA vasculitis in the long term, and rituximab was indicated here, but this would never have been given for GCA and could have led to irreversible kidney damage in someone who already only has a singular kidney.

**Key learning points:**

• The biggest learning point from this case in our opinion was the possibility of an underlying diagnosis for temporal arteritis which was not giant cell arteritis or another large vessel vasculitis, and instead was small to medium vessel vasculitis. This should prompt consideration to widen the net when looking for diagnoses in patients with the possibility of an alternative diagnosis.

• Despite this, adding ANCA tests to every patient with temporal arteritis would be counter productive with false positive results possible. However, there are some who would benefit from further investigation beyond the standard in a presentation o possible GCA.

• It may be the case that other mixed patterns of GCA and GPA are currently half treated in our patient populations, and in the future, it may be that more research on this topic will lead to identification of these patients and ensure that they are on the correct treatment pathways.

